# A systematic review and meta‐analysis of preoperative MRI‐based pelvimetry in predicting surgical difficulty in rectal cancer: Implications for personalised surgical planning

**DOI:** 10.1111/codi.70644

**Published:** 2026-10-05

**Authors:** Daichi Kitaguchi, Fiona R. Kolbinger, Paul Dipanjyoti, Kshitij Jadhav, Tatsuya Oda, Antonello Forgione, Jacques Marescaux

**Affiliations:** ^1^ Department of Surgery, Institute of Medicine University of Tsukuba Tsukuba Japan; ^2^ Research Institute against Digestive Cancer (IRCAD) Strasbourg France; ^3^ Weldon School of Biomedical Engineering Purdue University West Lafayette Indiana USA; ^4^ Department of Visceral, Thoracic and Vascular Surgery, Medical Faculty and University Hospital Dresden TUD Dresden University of Technology Dresden Germany; ^5^ Indian Institute of Engineering Science and Technology (IIEST) Shibpur India; ^6^ Koita Centre for Digital Health Indian Institute of Technology (IIT) Bombay Mumbai India

**Keywords:** magnetic resonance imaging, pelvimetry, rectal neoplasms, surgical difficulty, systematic review, total mesorectal excision

## Abstract

**Background:**

Magnetic resonance imaging (MRI)‐based pelvimetry has been investigated for predicting surgical difficulty in total mesorectal excision (TME) for rectal cancer. However, heterogeneity in pelvimetric parameters, surrogate outcomes, and study design has resulted in inconsistent evidence. This systematic review aimed to synthesise the evidence on commonly used MRI parameters and difficulty surrogates and assess their methodological robustness and clinical relevance.

**Methods:**

PubMed, Embase, and the Cochrane Library were systematically searched through December 2025 for studies evaluating preoperative MRI pelvimetry in patients undergoing TME for rectal cancer. Outcome parameters were surrogate measures of surgical difficulty, including operating time, intraoperative blood loss, conversion to open surgery, pathological quality metrics, and postoperative complications. Risk of bias was assessed using the Risk of Bias in Non‐randomised Studies of Exposures (ROBINS‐E) tool. This systematic review and meta‐analysis was reported in accordance with the PRISMA 2020 statement and prospectively registered (PROSPERO: CRD420261287153).

**Results:**

Thirty‐six studies comprising 8,364 patients were included. Common parameters included sagittal measurements and axial interspinous distance (ISD) and intertuberous distance (ITD). Definitions of surgical difficulty varied substantially. ROBINS‐E identified some concerns in 30 studies, a high risk of bias in five, and insufficient information in one. Meta‐analysis of six studies (*n* = 769) showed that shorter ISD (mean difference [MD] −5.13 mm, 95% CI −6.61 to −3.65; *p* < 0.001; *I* ^2^ = 0%) and ITD (MD −7.32 mm, 95% CI −10.24 to −4.39; *p* = 0.004; *I* ^2^ = 0%) were significantly associated with surrogate measures of surgical difficulty.

**Conclusions:**

MRI‐based pelvimetry, particularly ISD and ITD, may help predict surgical difficulty in TME. However, methodological heterogeneity limits broader quantitative synthesis and highlights the need for standardised pelvimetric protocols.

**What does this paper add to the literature?**

This systematic review synthesises heterogeneous evidence on MRI‐based pelvimetry for predicting surgical difficulty in total mesorectal excision. It identifies interspinous and intertuberous distances as the pelvimetric parameters most consistently associated with surrogate measures of surgical difficulty and highlights the need for standardised pelvimetric parameters and definitions of surgical difficulty.

## INTRODUCTION

Since the 1980s, total mesorectal excision (TME) has substantially improved oncological outcomes in rectal cancer surgery [1, 2]. Following the adoption of minimally invasive techniques, randomised controlled trials (RCTs) in the 2010s, including the COLOUR II and COREAN trials, demonstrated the non‐inferiority of laparoscopic TME compared with open surgery [3, 4, 5, 6, 7]. However, other RCTs involving more technically challenging cases, such as ACOSOG Z6051 and ALaCaRT, failed to demonstrate non‐inferiority based on composite measures of successful resection, including circumferential and distal margin status and completeness of TME [8, 9, 10, 11]. These findings reflect the technical limitations of conventional laparoscopic TME, particularly in patients with a high body mass index (BMI) or a narrow pelvis. Alternative approaches, including robotic TME and transanal total mesorectal excision (TaTME), were developed to address these limitations, and recent RCTs have demonstrated their non‐inferiority compared with conventional laparoscopic TME [12, 13, 14, 15].

Concurrently, neoadjuvant treatment strategies have evolved, shifting from conventional chemoradiotherapy (CRT) toward total neoadjuvant therapy (TNT), alongside growing interest in organ preservation [16, 17, 18, 19, 20]. Nevertheless, the 2025 update of the European Society for Medical Oncology (ESMO) rectal cancer guidelines emphasised that routine preoperative radiotherapy for all patients remains controversial, given the low risk of local recurrence reported in selected patients undergoing high‐quality TME [21]. Consequently, both the surgical approach and treatment strategy now require individualised, case‐by‐case decision‐making that accounts for anatomical complexity and technical difficulty.

Against this background, magnetic resonance imaging (MRI)‐based pelvimetry has been extensively investigated as a component of multifactorial preoperative risk assessment for predicting surrogate measures of surgical difficulty during TME for rectal cancer [22]. However, substantial heterogeneity in pelvimetric parameters, study designs, and the surrogate outcomes used to define surgical difficulty—which span intraoperative, postoperative, and pathological/oncological domains—has resulted in fragmented evidence. This fragmentation is further compounded by evolving surgical techniques and the emergence of artificial intelligence (AI)‐based image analysis. Therefore, this systematic review aimed to synthesise the existing evidence on MRI‐based pelvimetry by evaluating commonly used parameters and difficulty surrogates, assessing their methodological rigour and clinical relevance, and examining their applicability to contemporary surgical practice and future AI‐driven image analysis frameworks.

## MATERIALS AND METHODS

### Study design

This systematic review was reported in accordance with the Preferred Reporting Items for Systematic Reviews and Meta‐Analyses (PRISMA) 2020 guidelines [23]. A prospective protocol was registered in the International Prospective Register of Systematic Reviews (PROSPERO; registration number CRD420261287153) before data extraction.

### Search strategy

A comprehensive literature search was conducted in PubMed, Embase, and the Cochrane Library to identify relevant English‐language publications from database inception through December 2025. A core search strategy was developed using a combination of free‐text keywords and controlled vocabulary terms related to rectal cancer, MRI, pelvimetry‐related anatomical parameters, surgical procedures, and surgery‐related outcomes. The baseline free‐text search strategy was as follows: (rectal cancer OR rectal carcinoma*) AND (MRI OR “magnetic resonance”) AND (pelvis OR pelvic OR pelvimetry OR intertuberous OR interspinous OR “pelvic dimension” OR “pelvic anatomy” OR “mesorectal fat” OR “mesorectal area”) AND (surgery OR surgical) AND (“operative time” OR “blood loss” OR “postoperative complication” OR difficult* OR “surgical difficulty” OR conversion OR “circumferential resection margin” OR CRM OR “specimen quality” OR “TME quality”). This core strategy was subsequently adapted for each database by incorporating appropriate controlled vocabulary (e.g., Medical Subject Headings [MeSH] in PubMed and Emtree terms in Embase) and applying database‐specific syntax. The complete search strategies for all databases are provided in Table S1, and the final search was updated in December 2025.

### Eligibility criteria

Studies were considered eligible for inclusion if they met all of the following criteria:

1. involved adult patients undergoing TME for rectal cancer;
2. reported both preoperative MRI–based pelvimetry and surgery‐related outcomes reflecting surgical difficulty; and
3. were full‐text, peer‐reviewed articles published in English

1. used computed tomography (CT)‐based pelvimetry exclusively without reporting surgical (surrogate) outcomes;
2. evaluated exclusively long‐term oncological outcomes without reporting surgery‐related or surgical difficulty‐related outcomes; or
3. were non‐original publications (e.g., reviews, editorials, conference abstracts, or case reports) or lacked sufficient extractable methodological data.

Studies were excluded if they met any of the following criteria:

We restricted the imaging modality to MRI to minimise modality‐related heterogeneity and to enable assessment of both pelvic anatomy and relevant soft‐tissue structures.

### Study selection

All records retrieved from the search were screened by one author based on titles and abstracts using predefined eligibility criteria, with all screening decisions independently verified by a second author. Studies deemed potentially eligible proceeded to full‐text assessment. Although formal inter‐reviewer agreement statistics were not calculated, disagreements at any stage were resolved through discussion and consensus. Reasons for exclusion at the full‐text stage were documented. Full‐text assessment followed the same screening process used for title and abstract screening and was conducted manually.

### Risk‐of‐bias assessment

The risk of bias for all included studies was evaluated independently by two authors across all domains of the Risk of Bias in Non‐randomised Studies of Exposures (ROBINS‐E) tool [24]. ROBINS‐E was preferred over the Risk of Bias in Non‐randomised Studies of Interventions (ROBINS‐I) tool because the included studies examined MRI‐determined pelvic dimensions as inherent, non‐manipulable exposures rather than therapeutic interventions [25]. Any discrepancies were resolved through discussion, and consensus was achieved in all cases.

### Data extraction

Data extraction encompassed all preoperative MRI pelvimetric parameters derived from anatomical landmarks used to measure pelvic distances, angles, areas or volumes. For each study, all pelvimetric parameters included in the analyses were recorded. Surgery‐related outcomes used as surrogate measures of surgical difficulty were also extracted. When studies reported multiple outcomes, each outcome was extracted and evaluated independently. Additionally, outcomes demonstrating statistically significant associations with surgical difficulty surrogates were identified and extracted separately. For each included study, the statistical methods used to analyse associations between pelvimetric parameters and surgical outcomes were documented.

### Statistical analysis

Meta‐analyses were performed for homogeneous parameters, with results presented as forest plots. For continuous outcomes, the inverse variance method was applied, and results were expressed as mean differences (MDs) with 95% confidence intervals (CIs). Heterogeneity for each outcome was assessed using the chi‐squared (χ^2^) test (Cochran's Q) and inconsistency index (*I* ^2^). A χ^2^ *p* value of <0.10 or an *I* ^2^ value of >50% indicated substantial heterogeneity. A random‐effects model was used, with between‐study variance estimated using the restricted maximum likelihood (REML) method and CIs calculated using the Hartung–Knapp–Sidik–Jonkman (HKSJ) method. When continuous data were reported as medians with ranges or interquartile ranges (IQRs), means and standard deviations (SDs) were re‐estimated using previously described methods [26, 27]. To minimise conceptual heterogeneity across studies, meta‐analyses were restricted to a subset of studies that used composite outcomes as surrogate measures of surgical difficulty. These composite or predefined combinations of surrogate measures of surgical difficulty incorporated intraoperative and postoperative parameters and were conceptually based on the framework established by Escal et al. [28]. Although the specific components and thresholds varied across studies, studies were considered eligible for meta‐analysis when these measures represented broadly comparable clinical domains of surgical difficulty. Meta‐analyses were conducted using RevMan Web (Cochrane, London, UK).

## RESULTS

### Study selection

Figure 1 illustrates the PRISMA flow diagram of the study selection process. After screening, 36 articles met the eligibility criteria and were included in this systematic review (Table S2).

**FIGURE 1:**
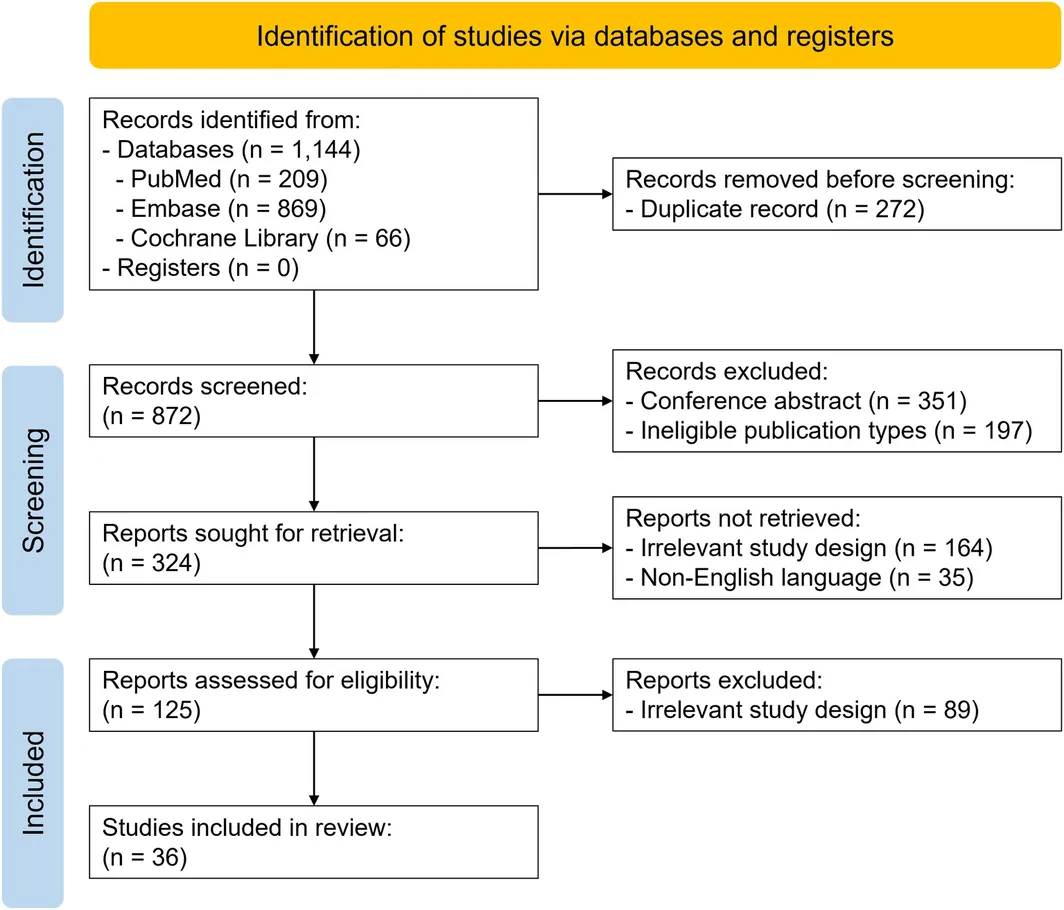
Preferred Reporting Items for Systematic Reviews and Meta‐Analyses (PRISMA) flow diagram of the study selection process.

### Study characteristics

Table 1 presents the characteristics of the 36 included studies, comprising 8,364 patients whose preoperative MRI data were analysed. Most studies were conducted in East Asia (19/36) and Europe (11/36). A total of 26 studies (72%) used a single‐centre design, whereas 10 (28%) were conducted in a multicentre setting. The inclusion periods were explicitly reported in 33 studies; among these, 11 (33%) were initiated in the 2000s, 20 (61%) in the 2010s, and two (6%) in the 2020s. Surgical approaches were reported in 33 studies: laparoscopic surgery was included in 26 (79%), followed by open surgery in 11 (33%) and robotic surgery in 10 (30%). TaTME was included in three studies (9%).

**TABLE 1:** Summary of characteristics of included studies.

Author and year	Country	Number of centres	Sample size	Study period	Eligibility criteria	Surgical approach	Analysed pelvimetric parameters	Surgical difficulty surrogate	Statistical method	Significant parameters
Atasoy 2018	Turkey	Single‐centre	125	Oct 2008–Feb 2011	AV ≤10 cm; cT3–4/N+; TME; nCRT	Open	PI, ITD, Pelvic depth, Interacetabular distance, S1/S3 distance, S3/Coccyx distance, Tumour volume	Operative time, Blood loss, CRM involvement, Anastomotic complications (AL)	Univariate analysis and multivariable linear and logistic regression	PI, ISD, Pelvic depth, Tumour volume
Ay 2025	Turkey	Single‐centre	52	Jan 2017–Sep 2022	AV ≤15 cm; TME; nCRT	Open, Laparoscopic	PI, ITD (ISD), Pelvic depth	Operative time, Blood loss, Length of hospital stay, Postoperative complications, Number of lymph nodes, TME quality	Univariate analysis and multivariable logistic regression	PI, ISD, Pelvic depth
Baek 2015	South Korea	Single‐centre	182	Jan 2008–Aug 2010	AV ≤15 cm	Robotic	PI, ISD, SCD, PO, ITD, Sacral depth	Operative time, Anastomotic complications, Postoperative complications, CRM involvement, Distal margin, Number of lymph nodes	Univariate analysis and multivariable linear regression	None
Baik 2008	South Korea	Single‐centre	100	May 2005–Mar 2006	AV ≤10 cm	Open	PI, ISD, SCD, Sacral depth, Transverse distance	TME quality, CRM involvement, Number of lymph nodes	Univariate analysis and multivariable logistic regression	PI, ISD
Baltus 2025	Netherlands	11	1707	Jan 2015–Dec 2021	Tumour below the sigmoidal take‐off	Laparoscopic, Robotic, TaTME	PI, SCD, PO, Sacrococcygeal angle	NA	Deep learning–based model (3D U‐Net)	NA (deep learning model)
Boyle 2005	UK	Single‐centre	126	Dec 1999–Jan 2002	AV ≤15 cm	Open	PI, ISD, SCD, PO, ITD, Pubic length, Pelvic midplane, Sacrococcygeal curve length, Sacrococcygeal curve area	CRM involvement	Univariate and correlation analysis	ISD, ITD
Cai 2023	China	Single‐centre	328	2016–2021	AV ≤10 cm; LAR with DST	Laparoscopic	Pelvic Morphology, Mesorectal Morphology, Tumour Morphology (Mask R‐CNN‐based segmentation)	Number of stapler cartridges	Deep learning–based model (3D CNN) with ROC analysis	NA (deep learning model)
Chau 2020	Canada	Single‐centre	92	2011–2017	AV ≤10 cm; LAR	Laparoscopic	PI, ISD, SCD, PO, ITD, Pubic length, Multiple pelvic angles, Pelvic midplane, AP distance, S1/5 distance	TME quality, CRM involvement, Distal margin, Operative time, Conversion to open	Multivariable linear and logistic regression	S1/S5/Pubic bottom angle
Chen 2021	China	Single‐centre	147	2015–2016	AV ≤10 cm; cT3–4/N+; LAR/ISR; nCRT	Laparoscopic	PI, ISD, SCD, PO, Pubic length, Sacral depth, Multiple pelvic angles, Pelvic depth, Mesorectal area, Rectal area	Operative time, Blood loss, Conversion to open, Transanal conversion, Postoperative complications, Length of hospital stay	Univariate analysis and multivariable logistic regression with nomogram	ISD, Pubic top/S1/S3 angle
de'Angelis 2018	France	4	170	Jan 2010–Jan 2016	AV ≤12 cm; cStage I–III; TME; nCRT	Laparoscopic	PI, ISD, SCD, PO, ITD, Pubic length, Multiple pelvic angles, Pelvic depth, Mesorectal area, Rectal area, S1/S3 distance, S3/Coccyx distance, Sacrococcygeal curve length, Sacrococcygeal curve area, Pelvic area, Interacetabular distance	Operative time, Blood loss, Conversion to open, Postoperative complication, Successful resection	Univariate analysis and multivariable regression	ISD, ITD, Pubic length
de'Angelis 2019	France	4	136	Jan 2010–Jan 2016	AV ≤12 cm; cStage II–III; LAR/ISR; nCRT	Laparoscopic	PI, ISD, SCD, PO, ITD, Pubic length, Multiple pelvic angles, Pelvic depth, Mesorectal area, Rectal area, S1/S3 distance, S3/Coccyx distance, Sacrococcygeal curve length, Sacrococcygeal curve area, Pelvic area, Interacetabular distance	Operative time, Blood loss, Conversion to open, Transanal conversion, Postoperative complications, Length of hospital stay	Univariate analysis and multivariable logistic regression	ISD
de'Angelis 2020	France	5	141	Feb 2016–Jun 2019	AV ≤12 cm; cStage II–III; LAR/ISR; nCRT	Laparoscopic	ISD	Operative time, Blood loss, Conversion to open, Transanal conversion, Postoperative complications, Length of hospital stay	Descriptive and comparative analysis with ROC analysis	ISD
Escal 2018	France	Single‐centre	164	2005–2014	AV ≤15 cm; LAR/ISR; nCRT	Open, Laparoscopic, Robotic	PI, ISD, SCD, PO, ITD, Mesorectal area, Rectal area, Transverse distance, Prostate volume	Operative time, Blood loss, Conversion to open, Transanal conversion, Postoperative complications, Length of hospital stay	Univariate analysis and multivariable logistic regression	ITD, Mesorectal area
Felsenreich 2023	USA	Multicenter	347	NR	Male; AV ≤6 cm; TME	Robotic	Mesorectal area	CRM involvement, Distal margin, TME quality, Number of lymph nodes, Operative time, Intraoperative and postoperative complications, Length of hospital stay, Mortality, Readmission, Reoperation	Univariate analysis and multivariable logistic regression	None
Ferko 2016	Czech Republic	Single‐centre	93	2011–2014	AV ≤10 cm; LAR/ISR	Open, Laparoscopic	PI, ISD, PO, ITD, Pubic length, Multiple pelvic angles, Transverse distance, S1/S3 distance, S3/Coccyx distance, Interacetabular distance	CRM involvement, Distal margin, TME quality	Univariate analysis and ordinal logistic regression	S1/Pubic top/Pubic bottom angle
Foo 2020	Hong Kong	Single‐centre	155	2013–2016	LAR with DST	Laparoscopic, Robotic	PI, ISD, SCD, PO, ITD	Number of stapler cartridges	Univariate analysis and multivariable linear and logistic regression	PI, ISD, ITD
Hong 2021	Australia	Multicentre	34	NR	TME	Open, Laparoscopic	ISD, SCD, ITD, Sacral depth, Rectal area, Pelvic area/volume, AP distance	CRM involvement	Univariate analysis and multivariable logistic regression	Pelvic volume
Huang 2025	China	Single‐center	264	Mar 2020–Mar 2024	Male; AV ≤7 cm; cStage I–III; LAR/ISR	Laparoscopic	S5/Anus/Urethral (SAU) angle	Operative time, Blood loss, TME quality, Anastomotic complications (AL)	Descriptive and univariate analysis	S5/Anus/Urethral (SAU) angle
Jiang 2025	China	2	1367	Jan 2018–Dec 2024	AV ≤10 cm; cT1–3; M0; LAR/ISR	Laparoscopic, Robotic	PI, ISD, SCD, PO, ITD, Pubic length, Sacral depth, Mesorectal area, Rectal area, Transverse distance, AP distance	Postoperative complications	Univariate analysis and multivariable logistic regression with machine learning–based models and nomogram	ISD, ITD
Killeen 2010	UK	Single‐centre	25	NR	TME	Laparoscopic	PI, ISD, PO, ITD, Multiple pelvic angles, S1/S3 distance, S3/Coccyx distance, Interacetabular distance	Operative time, CRM involvement	Descriptive and univariate correlation analysis	PI, ITD, PO, Sacrococcygeal angle, S3/Coccyx/Pubic bottom angle
Kim 2011	South Korea	Single‐centre	74	Apr 2008–Jul 2009	cT3–4/N+; M0; LAR with DST	Laparoscopic	PI, ISD, SCD, ITD, Sacral depth	Pelvic dissection time, Blood loss, Tumour perforation, TME quality	Univariate analysis and multivariable linear regression	SCD, ITD, Sacral depth
Kuo 2025	Taiwan	Single‐centre	527	Jan 2020–Dec 2023	AV ≤12 cm; LAR/ISR	Laparoscopic, Robotic	PI, ISD, SCD, PO, ITD, Pubic length, Sacral depth, Multiple pelvic angles, Pelvic depth, Mesorectal area, Transverse distance	Anastomotic complications	Univariate analysis and multivariable logistic regression with ROC analysis	ISD, Pubic top/S1/Coccyx angle
Lee 2020	South Korea	Single‐centre	85	Jan 2015–Dec 2015	AV ≤8 cm; cStage I–III; TME	Open, Laparoscopic, Robotic	Multiple pelvic floor muscle distances	Operative time	Univariate analysis and multivariable linear regression	Incline angle of pelvic floor muscle
Ma 2019	China	Multicenter	125	Jun 2010–Feb 2015	AV ≤12 cm; cStage II–III; LAR/ISR; nCRT/nCT	NR	Multiple pelvic muscle thicknesses, Rectal wall thickness, Distal sigmoid colon thickness, Mesorectal area	Anastomotic complications (AL)	Univariate analysis and multivariable regression with ROC analysis	Rectal wall thickness, Distal sigmoid colon thickness, Posterior mesorectal thickness
Ma 2022	China	Single‐centre	221	May 2007–Oct 2016	AV ≤8 cm; LAR/ISR	Open, Laparoscopic, TaTME	PI, ISD, SCD, PO, ITD, Pubic length, Multiple pelvic angles, Lateral pelvic floor muscle distance, Distance/angle from simulated trocar position, Transverse distance, AP distance, Prostate size, Tumour size	TME quality, Operative time, Blood loss, CRM involvement, Distal margin	Univariate analysis and multivariable logistic regression	ISD
McKechnie 2022	Canada	Single‐centre	83	Jan 2015–Dec 2019	cStage I–III; LAR	Laparoscopic, TaTME	ISD, Mesorectal area, Rectal area, Transverse distance, AP distance, Pelvic area	Operative time	Univariate analysis and multivariable linear regression	Posterior mesorectal thickness
Salerno 2006	UK	12	186	Jan 2002–Oct 2003	AV ≤16 cm; TME	NR	PI, ISD, SCD, PO, ITD, Pubic length, Multiple pelvic angles, Pelvic depth, S1/S3 distance, S3/Coccyx distance	Male sex (surrogate of surgical difficulty)	Univariate analysis and measurement reproducibility assessment	ISD, ITD
Salerno 2007	UK	12	186	Jan 2002–Oct 2003	AV ≤16 cm; TME	NR	PI, SCD, PO, Pubic length, Multiple pelvic angles, Pelvic depth, S1/S3 distance, S3/Coccyx distance	CRM involvement	Univariate analysis and measurement reproducibility assessment	None
Simpson 2020	UK	Single‐centre	122	Jan 2014–Dec 2017	TME	Open, Laparoscopic, Robotic	PI, ISD, SCD, PO, Pubic length, Sacral depth, Sacrococcygeal angle, Sacrococcygeal curve area	Specimen quality, CRM involvement, Operative time	Univariate analysis and multivariable linear and logistic regression	SCD, Sacrococcygeal angle
Sun 2021	China	Single‐centre	294	2014–2016	AV ≤10 cm; cT3–4/N+; LAR/ISR; nCRT	Laparoscopic	PI, ISD, SCD, PO, Pubic length, Sacral depth, Multiple pelvic angles, Pelvic depth, Mesorectal area, Rectal area	Operative time, Blood loss, Conversion to open, Transanal conversion, Postoperative complications, Length of hospital stay	Univariate analysis and multivariable logistic regression with nomogram development	ISD
Sun 2023	China	Single‐centre	108	Mar 2019–Oct 2022	AV ≤12 cm	Laparoscopic	Iliac bone morphology, Sacrococcygeal morphology (manual segmentation)	Surgeon‐rated difficulty, TME quality	Predictive score development followed by deep learning–based model validation	NA (deep learning model)
Teng 2022	China	Single‐centre	214	Jan 2018–Dec 2020	AV ≤10 cm; LAR/ISR	Laparoscopic	PI, ISD, SCD, PO, ITD, Sacral depth, Pelvic depth, Pubococcygeal distance, Anorectal angle, Sacrococcygeal angle	Operative time, Postoperative complications	Univariate analysis and multivariable logistic regression	PI, ITD, Pelvic depth
Tsuruta 2017	Japan	Single‐centre	43	Jul 2007–Jan 2013	Male; LAR with DST	Laparoscopic	PI, ISD, SCD, PO, ITD, Seminal vesicle/S4 distance, S4/Anus depth, Mesorectal area, Transverse distance, Rectal area	Anastomotic complications (AL)	Univariate analysis and multivariable regression with ROC analysis	ISD, Mesorectal thickness, S4/Anus depth
Yamamoto 2020	Japan	Single‐centre	121	Jul 2005–Jun 2017	AV ≤10 cm; LAR/ISR	Laparoscopic, Robotic	PI, ISD, SCD, PO, ITD, Sacral depth, Anorectal angle, Pubococcygeal distance	Operative time, Blood loss, Conversion to open, Transanal conversion, Postoperative complications, Length of hospital stay	Univariate analysis and multivariable logistic regression	PO, Anorectal angle
Yamaoka 2019	Japan	Single‐centre	98	Jan 2010–Dec 2016	LAR	Robotic	PI, ISD, SCD, PO, Pubic length, Sacral depth, Multiple pelvic angles, Mesorectal area, Rectal area, Transverse distance	Pelvic dissection time	Univariate analysis and multivariable linear regression	Mesorectal area
Yuan 2022	China	Single‐centre	122	Jun 2010–Aug 2021	AV ≤10 cm; cStage I–III; LAR/ISR	Open	PI, ISD, SCD, PO, ITD, Transverse distance	Operative time, Blood loss, Length of hospital stay, Postoperative complications	Univariate analysis and multivariable logistic regression with nomogram and ROC analysis	PI, ITD

Abbreviations: AL, anastomotic leakage; AP, anteroposterior; AV, anal verge; China, People's Republic of China; CRM, circumferential resection margin; DST, double‐stapling technique; ISD, interspinous distance; ISR, intersphincteric resection; ITD, intertuberous distance; LAR, low anterior resection; NA, not applicable; nCRT, neoadjuvant chemoradiotherapy; nCT, neoadjuvant chemotherapy; NR, not reported; PI, pelvic inlet; PO, pelvic outlet; ROC, receiver operating characteristic; SCD, sacrococcygeal distance; TaTME, transanal total mesorectal excision; TME, total mesorectal excision; UK, United Kingdom; USA, United States of America.

### Risk‐of‐bias assessment

The results of the risk‐of‐bias assessment using the ROBINS‐E tool are summarised in Figure 2. Five studies were classified as having a high risk of bias in the confounding domain (Domain 1) because of a failure to implement multivariable analyses or adjustment models for important clinical confounders, including sex, tumour height, or BMI. Most studies exhibited some concerns regarding bias in exposure measurement (Domain 2), primarily because of the manual nature of the pelvimetric measurements. Twenty‐six single‐centre studies were classified as having some concerns regarding bias in participant selection (Domain 3), primarily because of the limited representativeness inherent in single‐centre designs. Similarly, most studies raised some concerns regarding bias due to post‐exposure interventions (Domain 4), given the potential influence of intraoperative technical performance and decision‐making on surgical outcomes. Collectively, 30 studies were characterised as having some concerns, five as having a high risk of bias, and one as providing insufficient information, reflecting inherent structural limitations in observational pelvimetric research rather than suboptimal study conduct per se.

**FIGURE 2:**
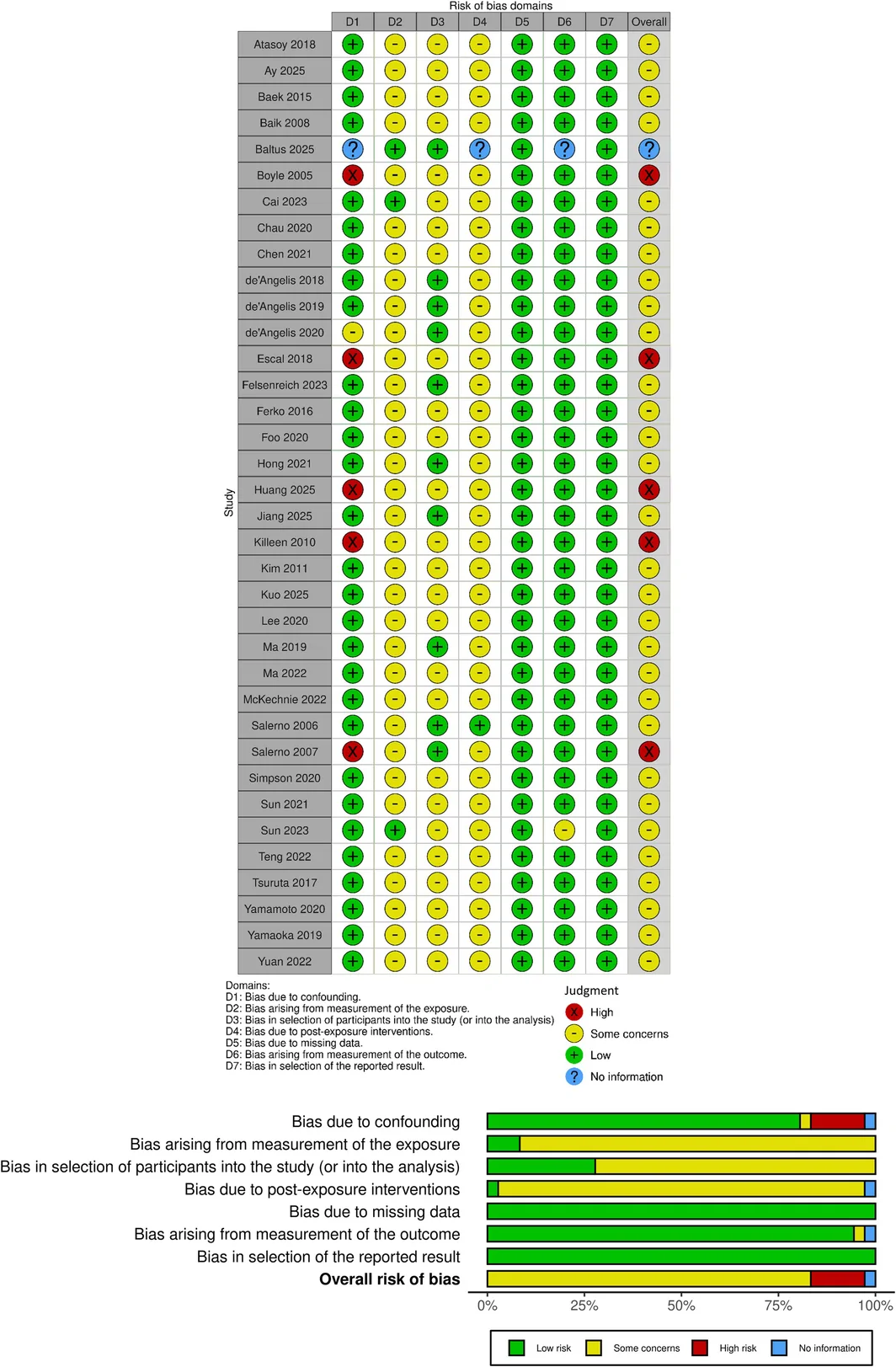
Results of the risk‐of‐bias assessment using the Risk of Bias in Non‐randomised Studies of Exposures (ROBINS‐E) tool.

### Pelvimetry and surgical difficulty metrics

The MRI‐based pelvimetric parameters evaluated across the 36 included studies are summarised in Figure 3A, with representative sagittal and axial MRI pelvimetric measurements shown in Figure 4. Sagittal plane measurements included pelvic inlet (PI) in 27 studies, sacrococcygeal distance (SCD) in 24 studies, pelvic outlet (PO) in 23 studies, pubic length in 14 studies, sacral depth in 12 studies, and pelvic depth in 10 studies (Figure 4A). Axial plane measurements included interspinous distance (ISD) in 27 studies (Figure 4B), intertuberous distance (ITD) in 21 studies (Figure 4C), transverse pelvic distance in 10 studies (Figure 4D), and area‐based parameters, including mesorectal area in 13 studies and rectal area in 10 studies (Figure 4B).

**FIGURE 3:**
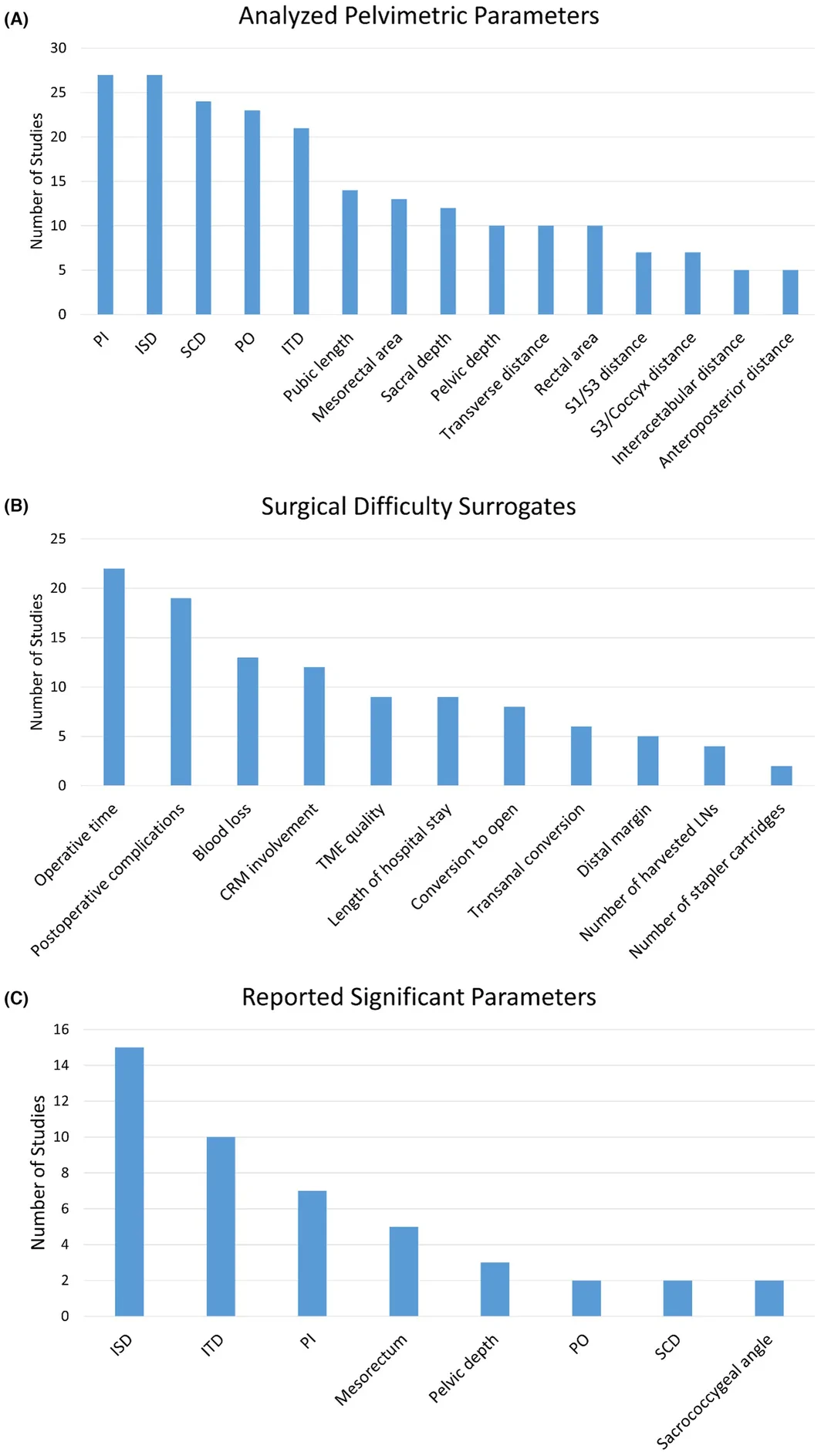
Results of the systematic review and evidence synthesis. (A) MRI‐based pelvimetric parameters evaluated in the included studies. (B) Surrogate outcomes used to represent surgical difficulty in the included studies. (C) MRI‐based pelvimetric parameters and the number of studies reporting significant associations with surrogate measures of surgical difficulty. PI, pelvic inlet; ISD, interspinous distance; SCD, sacrococcygeal distance; PO, pelvic outlet; ITD, intertuberous distance; CRM, circumferential resection margin; TME, total mesorectal excision; LN, lymph node.

**FIGURE 4:**
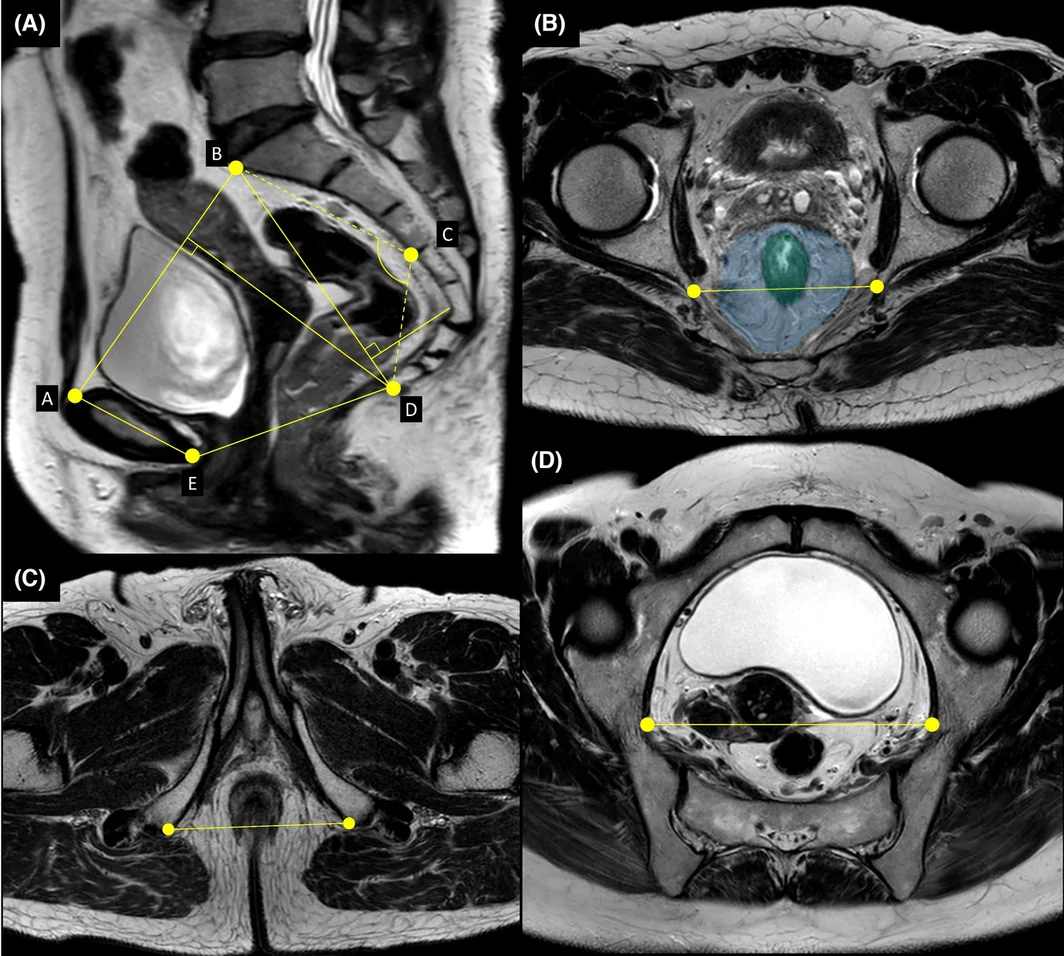
Representative examples of MRI‐based pelvimetric measurements in the sagittal and axial planes. (A) Sagittal plane measurements: Pelvic inlet (PI; distance between A and B), sacrococcygeal distance (SCD; distance between B and D), pelvic outlet (PO; distance between D and E), pubic length (distance between A and E), sacral depth (perpendicular distance from line AB to the deepest point of the sacrum), pelvic depth (perpendicular distance from line AB to point D), and sacrococcygeal angle (angle BCD). (B) Axial plane measurements: Yellow line, interspinous distance (ISD); blue area, mesorectal area; green area, rectal area. (C) Axial plane measurement of intertuberous distance (ITD). (D) Axial plane measurement of transverse pelvic distance.

The surrogate outcomes used to represent surgical difficulty across the 36 included studies are summarised in Figure 3B. Operating time, including total operating time and pelvic dissection time, was the most commonly reported surrogate outcome (22/36 studies). Other intraoperative outcomes were frequently assessed, including blood loss (13/36 studies), conversion to open surgery (8/36 studies), and conversion to transanal dissection (6/36 studies). Among the reported pathological outcomes, circumferential resection margin (CRM) involvement (12/36 studies) and TME quality (9/36 studies) were the most common. Postoperative outcomes, including global and anastomotic complications (19 studies) and length of hospital stay (9 studies), were also commonly evaluated.

Significant associations between pelvimetric parameters and surgical difficulty in individual studies are summarised in Figure 3C. ISD was most frequently reported to be significantly associated with surrogate measures of surgical difficulty (15/36 studies), followed by ITD (10/36 studies).

### Meta‐analysis

Results of the meta‐analysis are presented in Figure 5. The meta‐analysis included six studies comprising 769 patients that used composite measures reflecting surgical difficulty. These measures comprised intraoperative and postoperative parameters, including operating time, blood loss, conversion or additional surgical approaches, and postoperative outcomes such as hospital stay and morbidity. PI, PO, SCD, ISD and ITD were selected as MRI‐based pelvimetric parameters of interest. A shorter ISD was significantly associated with increased surgical difficulty (MD −5.13, 95% CI −6.61 to −3.65; *p* < 0.001), without substantial heterogeneity (*I* ^2^ = 0%). A shorter ITD was also significantly associated with increased surgical difficulty (MD −7.32, CI −10.24 to −4.39; *p* = 0.004), without substantial heterogeneity (*I* ^2^ = 0%). PI, PO and SCD were not significantly associated with surgical difficulty.

**FIGURE 5:**
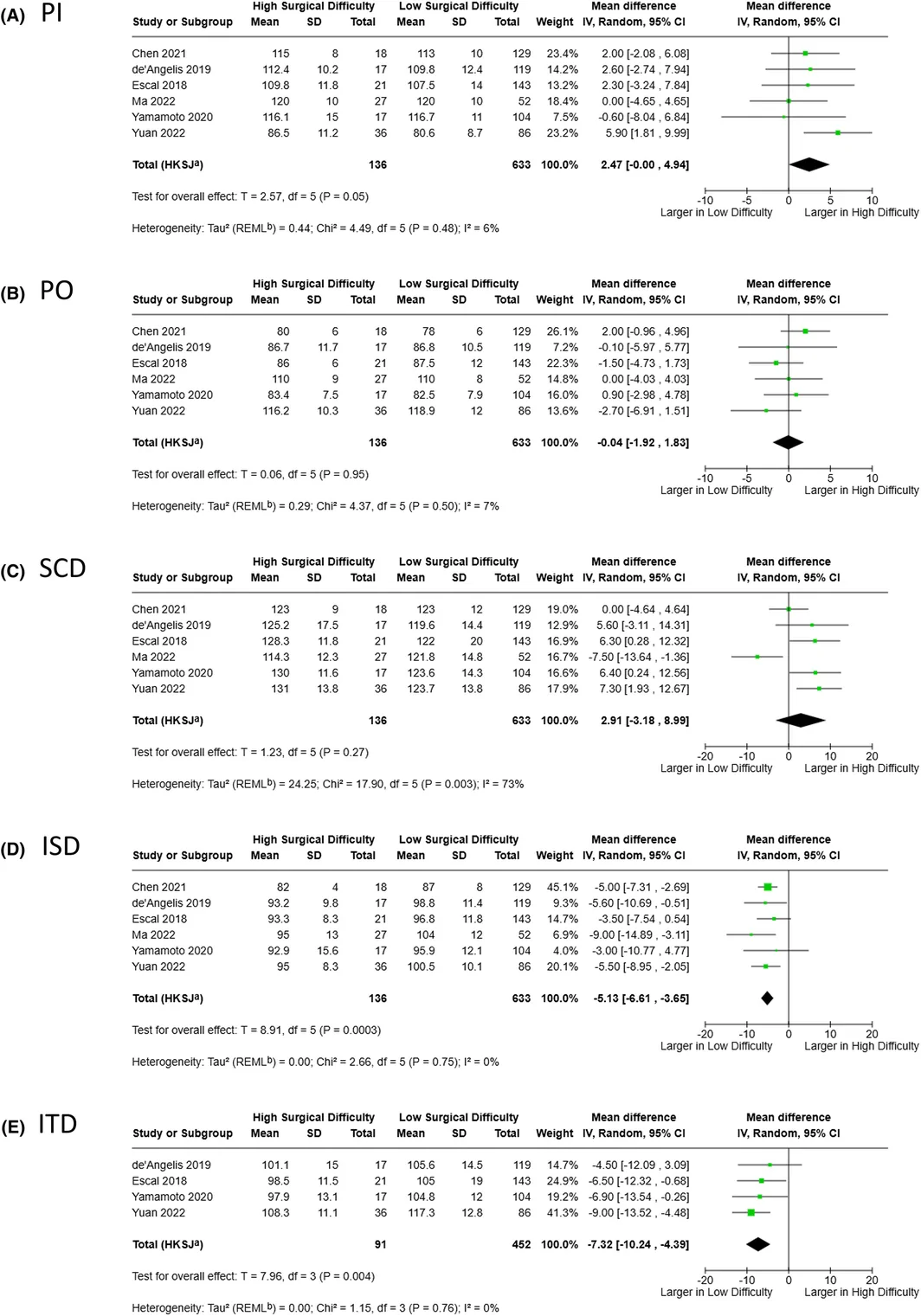
Forest plots of meta‐analysis results for MRI‐based pelvimetric parameters: (A) pelvic inlet (PI); (B) pelvic outlet (PO); (C) sacrococcygeal distance (SCD); (D) interspinous distance (ISD); (E) intertuberous distance (ITD).

## DISCUSSION

In this systematic review, we synthesised studies evaluating the ability of preoperative MRI‐based pelvimetry to predict surgical difficulty in rectal cancer surgery following TME. We comprehensively reviewed MRI‐based pelvimetric parameters and surrogate measures of surgical difficulty reported in the existing literature. Among the pelvimetric parameters, PI, ISD, SCD, PO and ITD were the most frequently adopted measures. Meta‐analysis demonstrated that shorter ISD and ITD, two frequently reported parameters, were significantly associated with greater surgical difficulty. Although numerous pelvimetric parameters have been proposed, no consensus has emerged regarding which measures are most informative. Our findings may help identify promising targets for future research. Notably, pelvimetric parameters in the majority of included studies were obtained manually, a practice known to introduce substantial inter‐ and intra‐observer variability. To overcome these limitations, the integration of advanced medical image analysis, particularly foundation models, may facilitate automated or semi‐automated measurements, thereby improving the reliability and reproducibility of pelvimetric assessments.

Surrogate measures of surgical difficulty varied widely, encompassing intraoperative metrics, postoperative clinical outcomes and pathological outcomes. This substantial heterogeneity indicates that no single measure adequately captures surgical difficulty. Instead, these findings support the development of composite measures that integrate multiple domains to more comprehensively reflect the multifaceted nature of surgical difficulty. Furthermore, composite outcomes could potentially be constructed using weighted averaging methods, with weights derived dynamically from frequently used indicators to reflect their relative informativeness.

In recent years, rectal cancer management has become increasingly complex with the availability of multiple surgical approaches and neoadjuvant treatment strategies. As treatment selection becomes increasingly individualised, accurate preoperative risk assessment has become more important. In this context, re‐examining the existing evidence on predictors of surgical difficulty is particularly relevant. Importantly, preoperative factors such as patient characteristics (e.g., age, sex and performance status) and preoperative therapies that may promote fibrosis or distort anatomical planes are potentially important determinants of surgical difficulty. These variables have not been adequately examined in previous studies and warrant further investigation in future surgical outcomes research.

Robotic TME and TaTME were introduced to address the limitations of conventional laparoscopic surgery, particularly the technical challenges posed by straight instruments and restricted manoeuvrability within a deep and narrow pelvis [29, 30, 31]. The articulated instruments of robotic systems and the bottom‐up perspective of the transanal approach both have the potential to mitigate pelvic anatomical constraints. In this review, no studies exclusively evaluated TaTME cohorts. Among studies limited to robotic TME, two of three did not demonstrate significant correlations between pelvimetric parameters and surrogate measures of surgical difficulty [32, 33, 34]. This observation suggests that robotic instrumentation may partially compensate for unfavourable pelvic anatomy; however, given the limited number of available studies, this finding should be interpreted with caution. Further data are required to clarify the role of pelvimetry in robotic TME and to determine whether similar or distinct patterns apply to TaTME.

A major source of potential bias identified in the included studies was the measurement of pelvimetric parameters. In most studies, distances, areas and angles were measured manually on MRI images. Such measurements are inherently operator‐dependent and susceptible to interobserver variability, potentially introducing measurement bias and limiting reproducibility across studies. AI‐based image analysis has emerged as a potential approach to address these limitations. Among the included studies, three employed deep learning‐based methods for MRI analysis [35, 36, 37]. Two of these studies used deep learning algorithms to automatically detect, segment and annotate target anatomical regions, thereby reducing reliance on manual landmark identification. These approaches may help standardise technically reproducible components of pelvimetry, including landmark detection, segmentation and geometric or volumetric measurements. However, the clinical interpretation of these measurements, particularly their relevance to surgical difficulty, will likely continue to require expert judgement.

Even with the identification of optimal pelvimetric parameters and the development of well‐structured composite surrogate outcomes, important limitations remain. Prediction models derived from single‐centre data sets are inherently limited in their generalisability and external validity and may not perform consistently when applied in other settings. In this review, 26 of the 36 included studies were single‐centre and were judged to have limited representativeness, reflecting an inherent limitation of this study design. Differences in patient characteristics, including obesity prevalence, MRI acquisition protocols and population‐level variation in pelvic morphology, may substantially affect model performance across institutional and geographic settings. Therefore, international multicentre studies are essential for developing robust and generalisable predictive models of surgical difficulty. The integration of diverse datasets across populations and imaging platforms will be critical for developing scalable and clinically applicable prediction models.

Finally, surgical outcomes are influenced by a combination of patient‐related factors and surgeon skill or technical efficiency. Although most included studies focused on patient‐level characteristics, intraoperative factors related to surgeon performance may also contribute substantially to patient outcomes and warrant systematic investigation in future studies. In addition, most studies employed conventional statistical approaches to evaluate associations between patient and treatment factors. However, these methods may not adequately capture complex interactions among variables that exert multiplicative effects on surgical outcomes. Machine learning techniques, paired with feature‐importance analyses, may better identify the most influential predictors of surgical outcomes. Such approaches may also enable the development of patient ‘personas’, defined by clusters of co‐occurring high‐impact features, which could serve as screening tools for personalised surgical risk prediction. Future prospective trials and multicentre registries could integrate MRI‐based pelvimetry, AI‐driven analytics and standardised surgical difficulty scores to enable harmonised risk stratification and externally validated prediction models.

This review had several limitations. First, because standardised definitions and outcome measures for surgical difficulty are lacking, direct quantitative comparison or prioritisation of individual pelvimetric parameters was not feasible. Although this review aimed to map the range of parameters and surrogate outcomes reported in the literature, such heterogeneity limits conclusions regarding their relative importance. These findings highlight the need for future consensus efforts, potentially using a structured Delphi process, to establish standardised definitions and outcome measures for surgical difficulty in TME for rectal cancer. Second, most included studies were retrospective and exploratory, precluding causal inference regarding the relationship between pelvic anatomy and surgical difficulty. Potential confounding factors, including surgeon experience, tumour characteristics and treatment strategies, were addressed inconsistently across studies. Third, the majority of studies were conducted at single centres, thereby limiting generalisability and external validity. Fourth, ongoing technological advances, including robotic surgery and AI‐based image analysis, may alter the relevance of traditional pelvimetric parameters over time, thereby complicating cross‐study interpretation.

Despite these limitations, this review provides a structured synthesis of heterogeneous pelvimetric parameters, surrogate outcomes and analytical approaches that may inform future research. Specifically, these findings provide a foundation for selecting appropriate pelvimetric parameters and outcome measures and for developing standardised, methodologically robust study designs to predict surgical difficulty.

## CONCLUSIONS

This systematic review demonstrated that ISD, ITD, PI, PO and SCD are the most frequently evaluated preoperative MRI‐based pelvimetric parameters for predicting surgical difficulty in TME for rectal cancer. Meta‐analysis demonstrated that shorter ISD and ITD were significantly associated with surrogate measures of surgical difficulty. Across the included studies, surgical difficulty was assessed using a wide range of surrogate outcomes, highlighting the need for well‐structured composite measures that integrate intraoperative metrics, postoperative clinical outcomes and pathological findings. Overall, this review synthesises the existing evidence to inform future study design and support the development of standardised pelvimetric assessments and outcome definitions for preoperative risk prediction, with potential implications for more individualised treatment strategies in rectal cancer.

## AUTHOR CONTRIBUTIONS

**Daichi Kitaguchi:** Conceptualization; methodology; writing – original draft. **Fiona R. Kolbinger:** Writing – review and editing. **Paul Dipanjyoti:** Conceptualization; methodology; writing – review and editing; funding acquisition. **Kshitij Jadhav:** Writing – review and editing; supervision. **Tatsuya Oda:** Writing – review and editing; supervision. **Antonello Forgione:** Writing – review and editing; supervision. **Jacques Marescaux:** Writing – review and editing; resources; supervision.

## FUNDING INFORMATION

This research was supported by the Japan Agency for Medical Research and Development (AMED) under Grant Number JP256f0137008. FRK receives support from the German Cancer Research Center (CoBot 2.0), the Central Indiana Corporate Partnership AnalytiXIN Initiative, the Evan and Sue Ann Werling Pancreatic Cancer Research Fund, and the Indiana Clinical and Translational Sciences Institute, funded in part by the National Institutes of Health, National Center for Advancing Translational Sciences, Clinical and Translational Sciences Award (UM1TR004402). The content is solely the responsibility of the authors and does not necessarily represent the official views of the National Institutes of Health. FRK declares an ongoing advisory role with the Surgical Data Science Collective, USA, and has received research funding from Novartis. All other authors declare that they have no relevant financial relationships.

## CONFLICT OF INTEREST STATEMENT

The authors declare that they have no competing interests related to this work.

## ETHICS STATEMENT

Not applicable. This study is a systematic review based on previously published data; therefore, ethical approval was not required.

## PATIENT CONSENT STATEMENT

Patient consent was not required because this study is a systematic review based exclusively on previously published data and does not involve individual patient data.

## PERMISSION TO REPRODUCE MATERIAL

No copyrighted material requiring permission for reproduction has been included in this manuscript.

## Supporting information


**Table S1:** Search strategies for each database.
**Table S2:** References of studies included in the systematic review.

**Data S1:** PRISMA 2020 Checklist.

## Data Availability

The data that support the findings of this study are available from the corresponding author upon reasonable request.

## References

[1] Heald RJ , Husband EM , Ryall RD . The mesorectum in rectal cancer surgery—the clue to pelvic recurrence? Br J Surg. 1982;69(10):613–616. 10.1002/bjs.1800691019 6751457

[2] Heald RJ , Ryall RD . Recurrence and survival after total mesorectal excision for rectal cancer. Lancet. 1986;1(8496):1479–1482. 10.1016/S0140-6736(86)91510-2 2425199

[3] van der Pas MH , Haglind E , Cuesta MA , Fürst A , Lacy AM , Hop WC , et al. Laparoscopic versus open surgery for rectal cancer (COLOR II): short‐term outcomes of a randomised, phase 3 trial. Lancet Oncol. 2013;14(3):210–218. 10.1016/S1470-2045(13)70016-0 23395398

[4] Bonjer HJ , Deijen CL , Abis GA , Cuesta MA , Van Der Pas MH , De Lange‐De Klerk ES , et al. A randomized trial of laparoscopic versus open surgery for rectal cancer. N Engl J Med. 2015;372(14):1324–1332. 10.1056/NEJMoa1414882 25830422

[5] Kang SB , Park JW , Jeong SY , Nam BH , Choi HS , Kim DW , et al. Open versus laparoscopic surgery for mid or low rectal cancer after neoadjuvant chemoradiotherapy (COREAN trial): short‐term outcomes of an open‐label randomised controlled trial. Lancet Oncol. 2010;11(7):637–645. 10.1016/S1470-2045(10)70131-5 20610322

[6] Jeong SY , Park JW , Nam BH , Kim S , Kang SB , Lim SB , et al. Open versus laparoscopic surgery for mid‐rectal or low‐rectal cancer after neoadjuvant chemoradiotherapy (COREAN trial): survival outcomes of an open‐label, non‐inferiority, randomised controlled trial. Lancet Oncol. 2014;15(7):767–774. 10.1016/S1470-2045(14)70205-0 24837215

[7] Park JW , Kang SB , Hao J , Lim SB , Choi HS , Kim DW , et al. Open versus laparoscopic surgery for mid or low rectal cancer after neoadjuvant chemoradiotherapy (COREAN trial): 10‐year follow‐up of an open‐label, non‐inferiority, randomised controlled trial. Lancet Gastroenterol Hepatol. 2021;6(7):569–577. 10.1016/S2468-1253(21)00094-7 33894918

[8] Fleshman J , Branda M , Sargent DJ , Boller AM , George V , Abbas M , et al. Effect of laparoscopic‐assisted resection vs open resection of stage II or III rectal cancer on pathologic outcomes: the ACOSOG Z6051 randomized clinical trial. JAMA. 2015;314(13):1346–1355. 10.1001/jama.2015.10529 PMC5140087 26441179

[9] Fleshman J , Branda ME , Sargent DJ , Boller AM , George VV , Abbas MA , et al. Disease‐free survival and local recurrence for laparoscopic resection compared with open resection of stage II to III rectal cancer: follow‐up results of the ACOSOG Z6051 randomized controlled trial. Ann Surg. 2019;269(4):589–595. 10.1097/SLA.0000000000003002 PMC6360134 30080730

[10] Stevenson AR , Solomon MJ , Lumley JW , Hewett P , Clouston AD , Gebski VJ , et al. Effect of laparoscopic‐assisted resection vs open resection on pathological outcomes in rectal cancer: the ALaCaRT randomized clinical trial. JAMA. 2015;314(13):1356–1363. 10.1001/jama.2015.12009 26441180

[11] Stevenson AR , Solomon MJ , Brown CS , Lumley JW , Hewett P , Clouston AD , et al. Disease‐free survival and local recurrence after laparoscopic‐assisted resection or open resection for rectal cancer: the Australasian laparoscopic cancer of the rectum randomized clinical trial. Ann Surg. 2019;269(4):596–602. 10.1097/SLA.0000000000003021 30247332

[12] Feng Q , Yuan W , Li T , Tang B , Jia B , Zhou Y , et al. Robotic versus laparoscopic surgery for middle and low rectal cancer (REAL): short‐term outcomes of a multicentre randomised controlled trial. Lancet Gastroenterol Hepatol. 2022;7(11):991–1004. 10.1016/S2468-1253(22)00248-5 36087608

[13] Feng Q , Yuan W , Li T , Tang B , Jia B , Zhou Y , et al. Robotic vs laparoscopic surgery for middle and low rectal cancer: the REAL randomized clinical trial. JAMA. 2025;334(2):136–148. 10.1001/jama.2025.8123 PMC12131176 40455621

[14] Liu H , Zeng Z , Zhang H , Wu M , Ma D , Wang Q , et al. Morbidity, mortality, and pathologic outcomes of transanal versus laparoscopic total mesorectal excision for rectal cancer short‐term outcomes from a multicenter randomized controlled trial. Ann Surg. 2023;277(1):1–6. 10.1097/SLA.0000000000005523 PMC9762710 35815886

[15] Zeng Z , Luo S , Zhang H , Wu M , Ma D , Wang Q , et al. Transanal vs laparoscopic total mesorectal excision and 3‐year disease‐free survival in rectal cancer: the TaLaR randomized clinical trial. JAMA. 2025;333(9):774–783. 10.1001/jama.2024.24276 PMC11880948 39847361

[16] Conroy T , Bosset JF , Etienne PL , Rio E , François É , Mesgouez‐Nebout N , et al. Neoadjuvant chemotherapy with FOLFIRINOX and preoperative chemoradiotherapy for patients with locally advanced rectal cancer (UNICANCER‐PRODIGE 23): a multicentre, randomised, open‐label, phase 3 trial. Lancet Oncol. 2021;22(5):702–715. 10.1016/S1470-2045(21)00079-6 33862000

[17] Conroy T , Castan F , Etienne PL , Rio E , Mesgouez‐Nebout N , Evesque L , et al. Total neoadjuvant therapy with mFOLFIRINOX versus preoperative chemoradiotherapy in patients with locally advanced rectal cancer: long‐term results of the UNICANCER‐PRODIGE 23 trial. Ann Oncol. 2024;35(10):873–881. 10.1016/j.annonc.2024.06.019 38986769

[18] Bahadoer RR , Dijkstra EA , van Etten B , Marijnen CA , Putter H , Kranenbarg EM , et al. Short‐course radiotherapy followed by chemotherapy before total mesorectal excision (TME) versus preoperative chemoradiotherapy, TME, and optional adjuvant chemotherapy in locally advanced rectal cancer (RAPIDO): a randomised, open‐label, phase 3 trial. Lancet Oncol. 2021;22(1):29–42. 10.1016/S1470-2045(20)30555-6 33301740

[19] Garcia‐Aguilar J , Patil S , Gollub MJ , Kim JK , Yuval JB , Thompson HM , et al. Organ preservation in patients with rectal adenocarcinoma treated with total neoadjuvant therapy. J Clin Oncol. 2022;40(23):2546–2556. 10.1200/JCO.22.00032 PMC9362876 35483010

[20] Verheij FS , Omer DM , Williams H , Lin ST , Qin LX , Buckley JT , et al. Long‐term results of organ preservation in patients with rectal adenocarcinoma treated with total neoadjuvant therapy: the randomized phase II OPRA trial. J Clin Oncol. 2024;42(5):500–506. 10.1200/JCO.23.01208 PMC11578087 37883738

[21] Hofheinz RD , Fokas E , Benhaim L , Price TJ , Arnold D , Beets‐Tan R , et al. Localised rectal cancer: ESMO clinical practice guideline for diagnosis, treatment and follow‐up. Ann Oncol. 2025;36(9):1007–1024. 10.1016/j.annonc.2025.05.528 40412553

[22] Hong JS , Brown KG , Waller J , Young CJ , Solomon MJ . The role of MRI pelvimetry in predicting technical difficulty and outcomes of open and minimally invasive total mesorectal excision: a systematic review. Tech Coloproctol. 2020;24(10):991–1000. 10.1007/s10151-020-02274-x 32623536

[23] Page MJ , McKenzie JE , Bossuyt PM , Boutron I , Hoffmann TC , Mulrow CD , et al. The PRISMA 2020 statement: an updated guideline for reporting systematic reviews. BMJ. 2021;372:n71. 10.1136/bmj.n71 PMC8005924 33782057

[24] Higgins JP , Morgan RL , Rooney AA , Taylor KW , Thayer KA , Silva RA , et al. A tool to assess risk of bias in non‐randomized follow‐up studies of exposure effects (ROBINS‐E). Environ Int. 2024;186:108602. 10.1016/j.envint.2024.108602 PMC11098530 38555664

[25] Sterne JA , Hernán MA , Reeves BC , Savović J , Berkman ND , Viswanathan M , et al. ROBINS‐I: a tool for assessing risk of bias in non‐randomised studies of interventions. BMJ. 2016;355:i4919. 10.1136/bmj.i4919 PMC5062054 27733354

[26] Wan X , Wang W , Liu J , Tong T . Estimating the sample mean and standard deviation from the sample size, median, range and/or interquartile range. BMC Med Res Methodol. 2014;14:135. 10.1186/1471-2288-14-135 PMC4383202 25524443

[27] Luo D , Wan X , Liu J , Tong T . Optimally estimating the sample mean from the sample size, median, mid‐range, and/or mid‐quartile range. Stat Methods Med Res. 2018;27(6):1785–1805. 10.1177/0962280216669183 27683581

[28] Escal L , Nougaret S , Guiu B , Bertrand MM , De Forges H , Tetreau R , et al. MRI‐based score to predict surgical difficulty in patients with rectal cancer. Br J Surg. 2018;105(1):140–146. 10.1002/bjs.10642 29088504

[29] Jayne D , Pigazzi A , Marshall H , Croft J , Corrigan N , Copeland J , et al. Effect of robotic‐assisted vs conventional laparoscopic surgery on risk of conversion to open laparotomy among patients undergoing resection for rectal cancer: the ROLARR randomized clinical trial. JAMA. 2017;318(16):1569–1580. 10.1001/jama.2017.7219 PMC5818805 29067426

[30] Deijen CL , Velthuis S , Tsai A , Mavroveli S , De Lange‐De Klerk ES , Sietses C , et al. COLOR III: a multicentre randomised clinical trial comparing transanal TME versus laparoscopic TME for mid and low rectal cancer. Surg Endosc. 2016;30(8):3210–3215. 10.1007/s00464-015-4615-x PMC4956704 26537907

[31] Serra‐Aracil X , Zarate A , Bargalló J , Gonzalez A , Serracant A , Roura J , et al. Transanal versus laparoscopic total mesorectal excision for mid and low rectal cancer (Ta‐LaTME study): multicentre, randomized, open‐label trial. Br J Surg. 2023;110(2):150–158. 10.1093/bjs/znac324 36224406

[32] Baek SJ , Kim CH , Cho MS , Bae SU , Hur H , Min BS , et al. Robotic surgery for rectal cancer can overcome difficulties associated with pelvic anatomy. Surg Endosc. 2015;29(6):1419–1424. 10.1007/s00464-014-3818-x 25159651

[33] Felsenreich DM , Gachabayov M , Bergamaschi R , RESURRECT Study Group . Does the mesorectal fat area impact the histopathology metrics of the specimen in males undergoing TME for distal rectal cancer? Updates Surg. 2023;75(3):581–588. 10.1007/s13304-022-01429-9 36513913

[34] Yamaoka Y , Yamaguchi T , Kinugasa Y , Shiomi A , Kagawa H , Yamakawa Y , et al. Mesorectal fat area as a useful predictor of the difficulty of robotic‐assisted laparoscopic total mesorectal excision for rectal cancer. Surg Endosc. 2019;33(2):557–566. 10.1007/s00464-018-6331-9 30006838

[35] Baltus SC , Geitenbeek RT , Frieben M , Thibeau‐Sutre E , Wolterink JM , Tan CO , et al. Deep learning‐based pelvimetry in pelvic MRI volumes for pre‐operative difficulty assessment of total mesorectal excision. Surg Endosc. 2025;39(3):1536–1543. 10.1007/s00464-024-11485-4 PMC11870868 39753930

[36] Cai ZH , Zhang Q , Fu ZW , Fingerhut A , Tan JW , Zang L , et al. Magnetic resonance imaging‐based deep learning model to predict multiple firings in double‐stapled colorectal anastomosis. World J Gastroenterol. 2023;29(3):536–548. 10.3748/wjg.v29.i3.536 PMC9850934 36688017

[37] Sun Z , Hou W , Liu W , Liu J , Li K , Wu B , et al. Establishment of surgical difficulty grading system and application of MRI‐based artificial intelligence to stratify difficulty in laparoscopic rectal surgery. Bioengineering (Basel). 2023;10(4):468. 10.3390/bioengineering10040468 PMC10135707 37106657

