# Supplementary material for: A systematic review and meta‐analysis of preoperative MRI‐based pelvimetry in predicting surgical difficulty in rectal cancer: Implications for personalised surgical planning
Source: Colorectal Dis. 2026 Oct 5;28(10):e70644. doi: 10.1111/codi.70644 (PMC13639373; doi:10.1111/codi.70644)
Supplement: Supplementary file 2 [file CODI-28-0-s002.docx]

**Supplementary Table 1:** Search strategies for each database

| Database | Date of last search | Search strategy |
| --- | --- | --- |
| PubMed | 2025-12-31 | ("Rectal Neoplasms"[Mesh] OR rectal cancer*[tiab] OR rectal carcinoma*[tiab])  AND ("Magnetic Resonance Imaging"[Mesh] OR MRI[tiab] OR "magnetic resonance"[tiab]) AND (pelvis[tiab] OR pelvic[tiab] OR pelvimetry[tiab] OR intertuberous[tiab] OR interspinous[tiab] OR "pelvic dimension*"[tiab] OR "pelvic anatomy"[tiab] OR "mesorectal fat"[tiab] OR "mesorectal area"[tiab]) AND ("Surgical Procedures, Operative"[Mesh] OR surgery[tiab] OR surgical[tiab]) AND ("Operative Time"[Mesh] OR "Blood Loss, Surgical"[Mesh] OR "Postoperative Complications"[Mesh] OR difficult*[tiab] OR "surgical difficulty"[tiab] OR conversion[tiab] OR "circumferential resection margin"[tiab] OR CRM[tiab] OR "specimen quality"[tiab] OR "TME quality"[tiab]) |
| Embase | 2025-12-31 | ('rectum cancer'/exp OR 'rectal cancer*':ti,ab OR 'rectal carcinoma*':ti,ab) AND ('magnetic resonance imaging'/exp OR mri:ti,ab OR 'magnetic resonance':ti,ab) AND (pelvis:ti,ab OR pelvic:ti,ab OR pelvimetry:ti,ab OR intertuberous:ti,ab OR interspinous:ti,ab OR 'pelvic dimension*':ti,ab OR 'pelvic anatomy':ti,ab OR 'mesorectal fat':ti,ab OR 'mesorectal area':ti,ab) AND ('surgery'/exp OR surgery:ti,ab OR surgical:ti,ab) AND ('operation time'/exp OR 'blood loss'/exp OR 'postoperative complication'/exp OR difficult*:ti,ab OR 'surgical difficulty':ti,ab OR conversion:ti,ab OR 'circumferential resection margin':ti,ab OR crm:ti,ab OR 'specimen quality':ti,ab OR 'tme quality':ti,ab) |
| Cochrane Library | 2025-12-31 | (rectal cancer* OR rectal carcinoma*) AND (MRI OR "magnetic resonance") AND (pelvis OR pelvic OR pelvimetry OR intertuberous OR interspinous OR "pelvic dimension" OR "pelvic anatomy" OR "mesorectal fat" OR "mesorectal area") AND (surgery OR surgical) AND ("operative time" OR "blood loss" OR "postoperative complication" OR difficult* OR "surgical difficulty" OR conversion OR "circumferential resection margin" OR CRM OR "specimen quality" OR "TME quality") |

**Supplementary Table 2:** References of studies included in the systematic review

| ID | Reference |
| --- | --- |
| 1 | Atasoy G, Arslan NC, Elibol FD, Sagol O, Obuz F, Sokmen S. Magnetic resonance-based pelvimetry and tumor volumetry can predict surgical difficulty and oncologic outcome in locally advanced mid-low rectal cancer. Surg Today. 2018 Dec;48(12):1040–1051. doi:10.1007/s00595-018-1690-3. |
| 2 | Ay OF, Firat D, Özçetin B, Ocakoglu G, Ozcan SGG, Bakır Ş, Ocak B, Taşkin AK. Role of pelvimetry in predicting surgical outcomes and morbidity in rectal cancer surgery: A retrospective analysis. World J Gastrointest Surg. 2025 Apr 27;17(4):104726. doi:10.4240/wjgs.v17.i4.104726. |
| 3 | Baek SJ, Kim CH, Cho MS, Bae SU, Hur H, Min BS, Baik SH, Lee KY, Kim NK. Robotic surgery for rectal cancer can overcome difficulties associated with pelvic anatomy. Surg Endosc. 2015 Jun;29(6):1419–1424. doi:10.1007/s00464-014-3818-x. |
| 4 | Baik SH, Kim NK, Lee KY, Sohn SK, Cho CH, Kim MJ, Kim H, Shinn RK. Factors influencing pathologic results after total mesorectal excision for rectal cancer: analysis of consecutive 100 cases. Ann Surg Oncol. 2008 Mar;15(3):721–728. doi:10.1245/s10434-007-9706-z. |
| 5 | Baltus SC, Geitenbeek RTJ, Frieben M, Thibeau-Sutre E, Wolterink JM, Tan CO, Vermeulen MC, Consten ECJ, Broeders IAMJ. Deep learning-based pelvimetry in pelvic MRI volumes for pre-operative difficulty assessment of total mesorectal excision. Surg Endosc. 2025 Mar;39(3):1536–1543. doi:10.1007/s00464-024-11485-4. |
| 6 | Boyle KM, Petty D, Chalmers AG, Quirke P, Cairns A, Finan PJ, Sagar PM, Burke D. MRI assessment of the bony pelvis may help predict resectability of rectal cancer. Colorectal Dis. 2005 May;7(3):232–240. doi:10.1111/j.1463-1318.2005.00819.x. |
| 7 | Cai ZH, Zhang Q, Fu ZW, Fingerhut A, Tan JW, Zang L, Dong F, Li SC, Wang SL, Ma JJ. Magnetic resonance imaging-based deep learning model to predict multiple firings in double-stapled colorectal anastomosis. World J Gastroenterol. 2023 Jan 21;29(3):536–548. doi:10.3748/wjg.v29.i3.536. |
| 8 | Chau J, Solomon J, Liberman AS, Charlebois P, Stein B, Lee L. Pelvic dimensions on preoperative imaging can identify poor-quality resections after laparoscopic low anterior resection for mid- and low rectal cancer. Surg Endosc. 2020 Oct;34(10):4609–4615. doi:10.1007/s00464-019-07209-8. |
| 9 | Chen J, Sun Y, Chi P, Sun B. MRI pelvimetry-based evaluation of surgical difficulty in laparoscopic total mesorectal excision after neoadjuvant chemoradiation for male rectal cancer. Surg Today. 2021 Jul;51(7):1144–1151. doi:10.1007/s00595-020-02211-3. |
| 12 | de'Angelis N, Pigneur F, Martínez-Pérez A, Vitali GC, Landi F, Torres-Sánchez T, Rodrigues V, Memeo R, Bianchi G, Brunetti F, Espin E, Ris F, Luciani A; EuMaRCS Study Group. Predictors of surgical outcomes and survival in rectal cancer patients undergoing laparoscopic total mesorectal excision after neoadjuvant chemoradiation therapy. Oncotarget. 2018 May 18;9(38):25315–25331. doi:10.18632/oncotarget.25431. |
| 10 | de'Angelis N, Pigneur F, Martínez-Pérez A, Vitali GC, Landi F, Gómez-Abril SA, Assalino M, Espin E, Ris F, Luciani A, Brunetti F; EuMaRCS Study Group. Assessing surgical difficulty in locally advanced mid-low rectal cancer: the accuracy of two MRI-based predictive scores. Colorectal Dis. 2019 Mar;21(3):277–286. doi:10.1111/codi.14473. |
| 11 | de'Angelis N, Martínez-Pérez A, Vitali GC, Pigneur F, Luciani A, Brunetti F, Kraft M, Martí-Martínez E, Moroni P, Espin E, Ris F, Pucciarelli S; EuMaRCS Study Group. Predicting Difficult Laparoscopic Total Mesorectal Excision for Locally-advanced Mid-low Rectal Cancer: The EuMaRCS Score Validation. Anticancer Res. 2020 Apr;40(4):2079–2087. doi:10.21873/anticanres.14166. |
| 13 | Escal L, Nougaret S, Guiu B, Bertrand MM, de Forges H, Tetreau R, Thézenas S, Rouanet P. MRI-based score to predict surgical difficulty in patients with rectal cancer. Br J Surg. 2018 Jan;105(1):140–146. doi:10.1002/bjs.10642. |
| 14 | Felsenreich DM, Gachabayov M, Bergamaschi R; RESURRECT Study Group. Does the mesorectal fat area impact the histopathology metrics of the specimen in males undergoing TME for distal rectal cancer? Updates Surg. 2023 Apr;75(3):581–588. doi:10.1007/s13304-022-01429-9. |
| 15 | Ferko A, Malý O, Örhalmi J, Dolejš J. CT/MRI pelvimetry as a useful tool when selecting patients with rectal cancer for transanal total mesorectal excision. Surg Endosc. 2016 Mar;30(3):1164–1171. doi:10.1007/s00464-015-4324-5. |
| 16 | Foo CC, Hung HT, Ho YC, Lam WWM, Law WL. Predicting the level of difficulty of the double-stapling technique in laparoscopic total mesorectal excision. Surg Endosc. 2020 Aug;34(8):3382–3387. doi:10.1007/s00464-019-07112-2. |
| 17 | Hong JSY, De Robles MS, Brown C, Brown KGM, Young CJ, Solomon MJ. Can MRI pelvimetry predict the technical difficulty of laparoscopic rectal cancer surgery? Int J Colorectal Dis. 2021 Dec;36(12):2613–2620. doi:10.1007/s00384-021-04000-x. |
| 18 | Huang X, Xiao Z, Huang Z. A simple and effective evaluation method to determine the difficulty of total mesorectal excision for male patients with mid and lower rectal cancer. Tech Coloproctol. 2025 Jul 19;29(1):145. doi:10.1007/s10151-025-03181-9. |
| 19 | Jiang Y, Baral S, Shi Y, Zhang Y, Fan P, Zhang X, Huang Y, Wang Y, Zhang H, Wang D. Developing and external validating a prediction model using machine learning and logistic regression. J Robot Surg. 2025 Dec 29;20(1):131. doi:10.1007/s11701-025-03015-8. |
| 20 | Killeen T, Banerjee S, Vijay V, Al-Dabbagh Z, Francis D, Warren S. Magnetic resonance pelvimetry as a predictor of difficulty in laparoscopic operations for rectal cancer. Surg Endosc. 2010 Dec;24(12):2974–2979. doi:10.1007/s00464-010-1075-1. |
| 21 | Kim JY, Kim YW, Kim NK, Hur H, Lee K, Min BS, Cho HJ. Pelvic anatomy as a factor in laparoscopic rectal surgery: a prospective study. Surg Laparosc Endosc Percutan Tech. 2011 Oct;21(5):334–339. doi:10.1097/SLE.0b013e31822b0dcb. |
| 22 | Kuo CY, Lee CY, Kung CH, Ngu JC, Kuo LJ. A predictive model for anastomotic complications after sphincter-preserving surgery for rectal cancer. Dis Colon Rectum. 2025 Nov;68(11):1296–1306. doi:10.1097/DCR.0000000000003938. |
| 23 | Lee JM, Han YD, Cho MS, Hur H, Min BS, Lee KY, Kim NK. Prediction of transabdominal total mesorectal excision difficulty according to the angle of pelvic floor muscle. Surg Endosc. 2020 Jul;34(7):3043–3050. doi:10.1007/s00464-019-07102-4. |
| 24 | Ma T, Zhong Q, Cao W, Qin Q, Meng X, Wang H, Wang J, Wang L. Clinical anastomotic leakage after rectal cancer resection can be predicted by pelvic anatomic features on preoperative MRI scans. Dis Colon Rectum. 2019 Nov;62(11):1326–1335. doi:10.1097/DCR.0000000000001481. |
| 25 | Ma Q, Cheng J, Bao Y, Gao Z, Jiang K, Wang S, Ye Y, Wang Y, Shen Z. Magnetic resonance imaging pelvimetry predicts the technical difficulty of rectal surgery. Asian J Surg. 2022 Dec;45(12):2626–2632. doi:10.1016/j.asjsur.2021.12.022. |
| 26 | McKechnie T, Ramji K, Kruse C, Jaffer H, Rebello R, Amin N, Doumouras AG, Hong D, Eskicioglu C. Posterior mesorectal thickness as a predictor of increased operative time. Surg Endosc. 2022 May;36(5):3520–3532. doi:10.1007/s00464-021-08674-w. |
| 27 | Salerno G, Daniels IR, Brown G, Heald RJ, Moran BJ. MRI pelvimetry in 186 patients with rectal cancer. Colorectal Dis. 2006 Nov;8(9):772–776. doi:10.1111/j.1463-1318.2006.01090.x. |
| 28 | Salerno G, Daniels IR, Brown G, Norman AR, Moran BJ, Heald RJ. Variations in pelvic dimensions do not predict CRM involvement. World J Surg. 2007 Jun;31(6):1313–1320. doi:10.1007/s00268-007-9007-5. |
| 29 | Simpson G, Marks T, Blacker S, Smith D, Walsh C. Sacrococcygeal dimensions and curvature are associated with resection quality. Tech Coloproctol. 2020 Oct;24(10):1063–1070. doi:10.1007/s10151-020-02273-y. |
| 30 | Sun Y, Chen J, Ye C, Lin H, Lu X, Huang Y, Chi P. Pelvimetric and nutritional factors predicting surgical difficulty. World J Surg. 2021 Jul;45(7):2261–2269. doi:10.1007/s00268-021-06080-w. |
| 31 | Sun Z, Hou W, Liu W, Liu J, Li K, Wu B, Lin G, Xue H, Pan J, Xiao Y. Establishment of surgical difficulty grading system and application of MRI-based AI. Bioengineering (Basel). 2023 Apr;10(4):468. doi:10.3390/bioengineering10040468. |
| 32 | Teng W, Liu J, Chen M, Zang W, Wu A. BMI and pelvimetry predict operative duration. BMC Surg. 2022 Nov 21;22(1):402. doi:10.1186/s12893-022-01840-4. |
| 33 | Tsuruta A, Tashiro J, Ishii T, Oka Y, Suzuki A, Kondo H, Yamaguchi S. Prediction of anastomotic leakage by pelvic measurement on MRI. Surg Laparosc Endosc Percutan Tech. 2017 Feb;27(1):54–59. doi:10.1097/SLE.0000000000000366. |
| 34 | Yamamoto T, Kawada K, Kiyasu Y, Itatani Y, Mizuno R, Hida K, Sakai Y. Prediction of surgical difficulty using MRI pelvimetry. BJS Open. 2020 Aug;4(4):666–677. doi:10.1002/bjs5.50292. |
| 35 | Yamaoka Y, Yamaguchi T, Kinugasa Y, Shiomi A, Kagawa H, Yamakawa Y, Furutani A, Manabe S, Torii K, Koido K, Mori K. Mesorectal fat area predicts difficulty of robotic TME. Surg Endosc. 2019 Feb;33(2):557–566. doi:10.1007/s00464-018-6331-9. |
| 36 | Yuan Y, Tong D, Liu M, Lu H, Shen F, Shi X. MRI-based pelvimetry nomogram. Front Oncol. 2022 Jul 25;12:882300. doi:10.3389/fonc.2022.882300. |
